# Seasonal Dynamics of Leaf Phenolic Compounds in *Rhododendron tomentosum* Harmaja Growing in a Coniferous Swamp Forest

**DOI:** 10.3390/plants15142195

**Published:** 2026-07-17

**Authors:** Martyna Vengrytė, Lina Raudonė

**Affiliations:** 1 Laboratory of Biopharmaceutical Research, Institute of Pharmaceutical Technologies, Lithuanian University of Health Sciences, Sukileliu Av. 13, LT-50162 Kaunas, Lithuania; lina.raudone@lsmu.lt; 2 Department of Pharmacognosy, Faculty of Pharmacy, Lithuanian University of Health Sciences, Sukileliu Av. 13, LT-50162 Kaunas, Lithuania

**Keywords:** *Rhododendron tomentosum*, phenolics, flavonoids, HPLC-PDA, phenological stage

## Abstract

*Rhododendron tomentosum* Harmaja is an evergreen *Ericaceae* species recognized as a rich source of biologically active phenolic compounds. However, information on seasonal variation in its non-volatile phenolics throughout the annual phenological cycle remains limited. Therefore, this study investigated the influence of phenological development on the phenolic composition of *R. tomentosum* leaves collected from a natural population in Lithuania between October 2023 and October 2024. Phenolic profiles were determined using a validated HPLC–PDA method. A total of 25 phenolic compounds were identified, comprising phenolic acids, flavan-3-ols, procyanidins, flavonoids, and coumarin derivatives. The highest total phenolic content was observed during autumn–winter dormancy, whereas the lowest levels occurred during fruit development. Procyanidins and phenolic acids predominated during dormancy, while flavonoids, particularly quercitrin, hyperoside, and isoquercitrin, accumulated during flowering. PCA revealed clear phenological grouping of samples and demonstrated distinct associations between dormancy and procyanidin-rich profiles and flowering and flavonoid accumulation. These findings highlight phenological stage as a key factor influencing phenolic composition and indicate optimal harvesting periods depending on the target phenolic compounds.

## 1. Introduction

Wild rosemary (*Rhododendron tomentosum* Harmaja) is an evergreen shrub widely distributed in boreal regions of Europe, Asia and North America. The species is typically found in peatlands, bogs and swampy coniferous forests, where acidic soils, high moisture and low nutrient availability create specific environmental conditions for plant growth [1]. In these habitats, *R. tomentosum* forms part of characteristic peatland vegetation communities together with other acidophilic species such as *Calluna vulgaris*, *Andromeda polifolia* and *Vaccinium uliginosum*, while the tree layer is usually represented by scattered birch (*Betula* spp.) and small pine (*Pinus sylvestris*) individuals [2].

Plants growing in peatland ecosystems are often exposed to environmental stressors such as nutrient limitation, high soil acidity, and water-saturated substrates. As a result, many peatland species accumulate considerable amounts of phenolic compounds, which play an important role in plant defense and physiological regulation [3]. Phenolic compounds are known to contribute to protection against oxidative stress, ultraviolet radiation, herbivores and microbial pathogens, and they are also widely studied due to their biological activities, including antioxidant, antimicrobial, and anti-inflammatory effects [4].

Species belonging to the *Ericaceae* family, such as *R. tomentosum*, are particularly rich in phenolic metabolites. Previous phytochemical studies have reported the presence of phenolic acids, flavan-3-ols, procyanidins and flavonol glycosides in different *Ericaceae* species [5,6]. Flavonol glycosides such as rutin, hyperoside, isoquercitrin and quercitrin are commonly detected in leaves of *Ericaceae* plants and are considered characteristic compounds of this plant family [5].

As an evergreen species, *R. tomentosum* retains its leaves throughout the year. In this evergreen species, phenological changes are mainly related to shoot development, bud formation, flowering, and fruit maturation rather than leaf emergence, typical of deciduous plants. Nevertheless, seasonal changes in plant metabolism may still lead to variation in the accumulation of secondary metabolites during different stages of plant development.

Although the phytochemistry of *R. tomentosum* has been investigated in several studies, most research has focused primarily on essential oil composition and general chemical characterization of the species [7,8,9,10]. Few studies have examined seasonal variation in non-volatile phenolics of *R. tomentosum*, and full annual phenological-cycle data from European natural populations remain limited.

In recent years, increasing attention has been given to the phytochemical composition of forest plants due to their ecological importance and potential applications as sources of biologically active compounds [11]. Phenolic metabolites represent one of the most diverse classes of plant secondary metabolites and are widely investigated for their antioxidant, antimicrobial and anti-inflammatory properties [12]. Forest ecosystems, including peatlands and swampy coniferous forests, are considered important reservoirs of such bioactive compounds, as plants growing in these environments often produce elevated levels of secondary metabolites as adaptive responses to environmental stress conditions [2,3]. Investigating seasonal rhythms in phenolic compounds accumulation and broader phytochemical composition is crucial because temporal variation can substantially influence experimental outcomes, interpretation of plant bioactivity, understanding of regulatory mechanisms, the quality, standardization, and efficacy of phytochemical products.

To date, available seasonal studies on *R. tomentosum* have mainly addressed the snow-free growing season or selected phenolic constituents, while the annual dynamics of non-volatile phenolics in European natural populations remain insufficiently characterized. The present study provides results on the full annual phenological phytochemical analysis of *R. tomentosum* leaves, which, to our knowledge, was not performed before. This study design enabled the identification of compound-specific phenological patterns and compounds associated with particular phenological stages, as well as the definition of optimal harvesting periods for target compounds. In particular, the inclusion of winter dormancy and microphenological stages provides new information that cannot be obtained from conventional spring–summer sampling design.

Therefore, the aim of the present study was to investigate the variation in phenolic compound composition in *R. tomentosum* leaves collected throughout the annual period in a swampy coniferous forest habitat that has not been determined previously for European natural populations of this species, and to evaluate the influence of phenological development on the accumulation of these compounds.

## 2. Results

### 2.1. Phenolic Compound Profile of R. tomentosum

Chromatographic analysis of *Rhododendron tomentosum* leaf samples revealed a complex phenolic profile consisting of flavonols, flavan-3-ols, proanthocyanidins, coumarins, and phenolic acid derivatives (Table 1, Table 2 and Table 3).

The phenolic composition of *R. tomentosum* leaves varied considerably throughout the vegetation period, reflecting clear phenological stage-dependent metabolic changes. Quantitative analysis revealed the presence of phenolic acids, flavan-3-ols, procyanidin derivatives, flavonoids, and coumarins, with concentrations differing significantly among sampling points (*p* < 0.05).

The total identified phenolic content ranged from 32,765.71 µg/g DW (DT15; early fruit development stage) to 72,308.20 µg/g DW (DT3; winter dormancy stage). In general, leaf samples collected during the autumn–winter period (October–February; DT1–DT6) contained the highest total phenolic levels, whereas samples collected during spring and summer (March–August; DT7–DT17) exhibited substantially lower totals. A gradual decline in total phenolic content was observed from dormancy through active growth and reproductive development.

When grouped according to phenological stage, the mean total identified phenolic content was highest during autumn–winter dormancy, followed by bud development, flowering, and fruiting stages, confirming a progressive reduction in the overall phenolic content toward later developmental stages. Differences between dormancy and subsequent stages were statistically significant (*p* < 0.05).

A more detailed analysis revealed that most individual compounds exhibited statistically significant seasonal variation (*p* < 0.001). This was further confirmed by Tukey’s HSD test, which showed distinct grouping of sampling periods for most compounds, indicating clear differences between autumn–winter, flowering, and fruit development stages. The phenolic profile was dominated by chlorogenic acid (up to 16,339.2 µg/g DW), procyanidin derivatives (up to 8323 µg/g DW), neochlorogenic acid (up to 8744 µg/g DW), avicularin (up to 6177 µg/g DW), and procyanidin A1 (up to 5809 µg/g DW), which together constituted the major proportion of the total phenolic content.

A pronounced decreasing trend from dormancy to fruiting was observed for flavan-3-ols and procyanidin-type compounds, including procyanidin derivatives, procyanidin A1, epicatechin/procyanidin trimer, and the chlorogenic acid/catechin complex (*p* < 0.001). According to the post hoc grouping, autumn–winter samples were generally assigned to the highest concentration groups for these compounds, whereas flowering and fruit development samples were grouped among the lower concentration levels. In contrast, flavonoids exhibited an opposite pattern. Quercitrin showed a pronounced increase during the flowering stage (DT13–DT14), reaching up to approximately 19,517 µg/g DW (*p* < 0.001). Similarly, hyperoside and isoquercitrin displayed elevated levels during flowering. Tukey’s HSD test confirmed that flowering stages formed distinct high-concentration groups for these flavonoids compared with most other sampling periods, suggesting increased flavonoid biosynthesis associated with reproductive development.

Some compounds remained relatively stable throughout the vegetation period. Notably, neochlorogenic acid (*p* = 0.471) and guaijaverin (*p* = 0.479) did not show statistically significant variation, suggesting their potential role as constitutive metabolites that maintain baseline physiological functions. This was also confirmed by Tukey’s HSD test, where all sampling periods were assigned to the same post hoc group for these compounds.

Based on their seasonal dynamics, several compounds were closely associated with specific phenological stages. Flavan-3-ols and procyanidin derivatives accumulated predominantly during autumn–winter stages, whereas flavonoids such as quercitrin, hyperoside, and isoquercitrin reached their highest concentrations during flowering. In contrast, neochlorogenic acid and guaijaverin remained relatively stable throughout the annual cycle, showing only minor seasonal variation.

An additional noteworthy observation was the difference between the two samples collected in March (DT8 and DT9), representing brown and green bud stages. Although sampled on the same date, they exhibited distinct phenolic fingerprints, indicating that microphenological status may influence metabolite composition independently of collection date. This finding highlights the importance of detailed phenological characterisation when interpreting seasonal phytochemical dynamics in *R. tomentosum* leaves.

**Figure 1:**
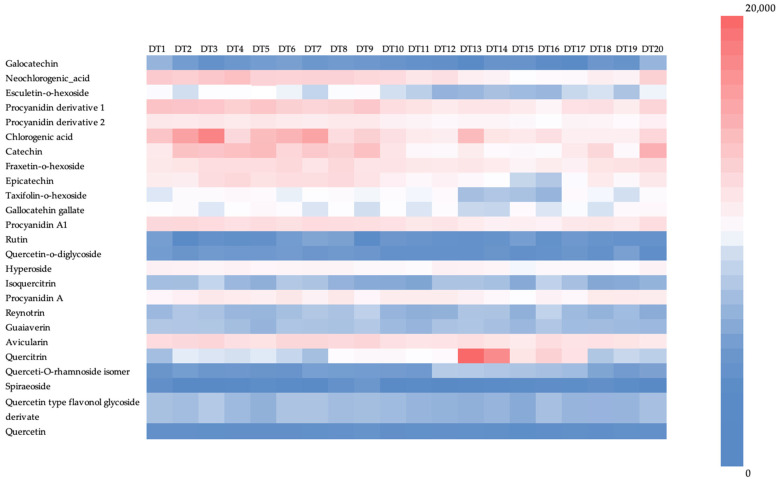
Heatmap of phenolic compound variation in *R. tomentosum* leaves across phenological stages. Red indicates higher concentrations, while blue represents lower values. The meaning of coding is explained in Section 4.2.

To better visualize the variation in phenolic composition across phenological stages, a heatmap was constructed (Figure 1).

A more detailed analysis of phenolic composition revealed distinct differences in the seasonal dynamics of individual compound groups in *R. tomentosum* leaves.

Flavan-3-ol compounds, including gallocatechin, epicatechin, and gallocatechin gallate, showed clear seasonal variation throughout the vegetation period. Among these, epicatechin remained the dominant compound, while gallocatechin and its gallate derivative were present in lower amounts. The total flavan-3-ol content followed a pronounced phenological pattern, with higher levels observed during autumn, winter, and early spring (DT1–DT8), reaching peak values of approximately 7173.41 µg/g. A gradual decrease occurred during active vegetative growth and flowering (DT10–DT16), where the lowest concentrations (~1722.07 µg/g) were recorded, followed by a subsequent increase during late vegetation stages (DT19–DT20).

A similar but more pronounced trend was observed for procyanidin-type compounds, which constituted the most abundant phenolic group identified in the extracts. Their total content reached a maximum of 21,424.39 µg/g during autumn–winter stages (DT3) and decreased continuously toward summer (DT10–DT16), reaching the lowest levels (8637.42 µg/g). This was followed by a gradual increase during late vegetation stages (DT17–DT20), suggesting a strong seasonal pattern and a potential role in environmental stress adaptation.

Phenolic acids, mainly represented by chlorogenic and neochlorogenic acids, also contributed substantially to the overall phenolic pool. Their total content showed high values during autumn and winter (up to 24,178.50 µg/g at DT3), followed by a decrease toward spring and early summer (DT10–DT15), where the lowest levels (~5319.50 µg/g) were observed. Toward the end of the vegetation period, phenolic acid levels increased again (up to 12,811.70 µg/g at DT20), indicating a seasonal redistribution pattern similar to that observed for procyanidins.

On the other hand, flavonoids exhibited a different seasonal profile. This group, including taxifolin-O-hexoside, rutin, quercetin-O-diglycoside, hyperoside, isoquercitrin, reynoutrin, guaijaverin, avicularin, quercitrin, quercetin-3-O-rhamnoside derivative, spiraeoside, quercetin-glycoside derivative, was consistently present across all samples, indicating a relatively stable occurrence throughout the vegetation cycle. The total flavonoid content ranged from 10,361.20 µg/g (DT18) to 25,426.69 µg/g (DT13), with the highest levels observed during the flowering period (DT13–DT14). Across all collection dates, avicularin contributed the most to the total identified flavonoids (16–44%), followed by quercitrin and hyperoside. The relative importance of individual compounds changed strongly with phenological development. In particular, quercitrin accumulation increased significantly before flowering and became more predominant during flowering and fruit development stages, up to 34 and 39% of total identified flavonoids, respectively.

In contrast to flavan-3-ols and procyanidins, flavonoids did not exhibit a strong decline during active growth stages but instead showed increased accumulation during flowering, suggesting a closer association with reproductive processes.

Coumarin derivatives, including esculetin-O-hexoside and fraxetin-O-hexoside, were present in lower amounts compared to other phenolic groups. Their total content showed moderate variation throughout the vegetation period, ranging from approximately 2977.56 µg/g to 6920.09 µg/g, with higher levels observed during autumn and early spring (DT3–DT8) and lower concentrations during late spring and summer (DT12–DT15).

To complement the chromatographic characterization of individual compounds and to provide an integrated assessment of major phenolic groups, spectrophotometric assays were additionally performed for total phenolics, flavonoids, phenolic acids, and proanthocyanidins. Spectrophotometric analysis revealed seasonal variation in all investigated phenolic groups (Table 4). Total phenolic content ranged from 26.10 to 53.80 mg GAE/g DW, flavonoid content ranged from 11.40 to 32.40 mg RE/g DW, phenolic acid content ranged from 82.50 to 165.48 mg CRE/g DW, and proanthocyanidins from 1.15 to 3.23 mg EE/g DW. In agreement with HPLC-PDA results, the highest concentrations were observed during autumn–winter dormancy, whereas flowering and fruit development stages were characterized by substantially lower levels.

Correlational interrelationships were tested between the meteorological conditions (Table 5) registered and the amounts of phenolic compounds. Average air temperatures showed strong negative correlations with total flavan-3-ols (ρ = −0.819, *p* < 0.001), phenolic acids (ρ = −0.791, *p* < 0.001), coumarins (ρ = −0.784, *p* < 0.001), and proanthocyanidins (ρ = −0.864, *p* < 0.001). In contrast, no significant correlation was determined for the total flavonoids. Precipitation showed no significant correlations with the analysed phenolic groups. These results indicate that the seasonal accumulation of phenolic acids, flavan-3-ols, coumarins, and particularly proanthocyanidins was associated mainly with lower temperatures and, for coumarins and proanthocyanidins, with higher humidity (ρ = 0.511, *p* = 0.021, and ρ = 0.604, *p* = 0.005, respectively) and cloudiness (ρ = 0.587, *p* = 0.007, and ρ = 0.505, *p* = 0.023, respectively), whereas flavonoid accumulaton followed a different pattern strongly dependent on individual ontogenesis.

Similar environmental associations were observed for spectrophotometrically determined phenolic groups. Average air temperature was negatively associated with phenolic acids (ρ = −0.713, *p* < 0.001), flavonoids (ρ = −0.529, *p* = 0.017), and proanthocyanidins (ρ = −0.646, *p* = 0.002), whereas relative humidity showed positive correlations with these compound groups (ρ = 0.527, *p* = 0.017; ρ = 0.485, *p* = 0.030; and ρ = 0.581, *p* = 0.007, respectively). Furthermore, total phenolic content correlated strongly with phenolic acids (ρ = 0.762, *p* < 0.001) and proanthocyanidins (ρ = 0.879, *p* < 0.001), suggesting that these compounds were the major contributors to seasonal variation in total phenolic content.

### 2.2. Principal Component Analysis (PCA)

A principal component analysis (PCA) was performed in order to evaluate overall variation in phenolic composition across different phenological stages of *R. tomentosum* leaf extract. The analysis was based on the total content of major phenolic groups, including phenolic acids, procyanidins, and flavonoids. PCA revealed four distinct sample clusters corresponding to autumn–winter dormancy, mass flowering, fruit development and late vegetation stages.

The PCA plot revealed a clear tendency of sample grouping according to phenological development. Early-stage samples (DT1–DT6) were clustered closely together in the upper-left region of the plot, indicating a similar phenolic composition during autumn and winter periods.

In contrast, samples representing later phenological stages were more dispersed and occupied the lower-left region of the score plot. However, flowering-stage samples (DT13–DT14) were clearly separated from the rest, indicating a distinct phenolic profile during this period.

The first two principal components (PC1 and PC2) explained 71.89% and 26.91% of the total variance, respectively, accounting for a cumulative 98.80% of the dataset variability. PC1 was mainly associated with phenolic acids and proanthocyanidins. Samples collected during autumn–winter dormancy were positioned on the positive side of PC1 and were associated with higher contents of phenolic acids and proanthocyanidins. These phenolic groups contributed most strongly to the separation of DT1–DT7 from spring–summer samples. PC2 was mainly associated with total flavonoid content. Flowering stage samples, DT13–DT14, were clearly separated from the other samples and were associated with increased total flavonoid levels. Samples collected during fruit development (DT15–DT18) were positioned away from the dormancy and flowering cluster, reflecting reduced overall phenolic accumulation. Their position on the negative sides of both PCs’ axes indicates lower total phenolic accumulation and a less pronounced dominance of any single phenolic group.

The relationships between the analysed phenolic groups were further clarified by examining the component loadings. According to the rotated component matrix, phenolic acids (0.979) and procyanidins (0.954) showed strong positive loadings on PC1, together with the total phenolic content (0.981), indicating a close positive association between these variables.

In contrast, flavonoids were strongly associated with PC2 (0.998) and showed only a weak contribution to PC1 (−0.062), suggesting a distinct and independent variation pattern compared to procyanidins and phenolic acids.

**Figure 2:**
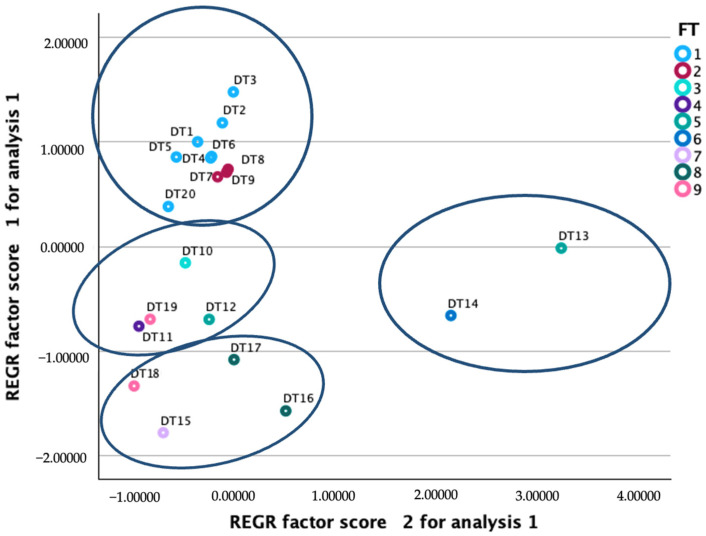
Principal Component Analysis (PCA) Score Plot Demonstrating Phenological Grouping of *R. tomentosum* Samples Based on Phenolic Profiles.

The opposite signs and separation of loadings between PC1 and PC2 indicate a clear metabolic differentiation between procyanidin/phenolic acid-dominated profiles and flavonoid-rich composition. This pattern is consistent with the observed seasonal trends, where procyanidins and phenolic acids were predominant during dormancy, whereas flavonoids were more closely associated with the flowering stage, Figure 2.

## 3. Discussion

Phenological stage is an important determinant of plant secondary metabolism, and the concentrations of individual phenolic compounds often change significantly throughout the vegetation cycle [13,14,15,16]. The results demonstrate that phenological development strongly influences the phenolic qualitative and quantitative composition of *R. tomentosum* leaves. The environmental conditions and developmental processes can significantly influence secondary metabolite accumulation in plants [16,17,18]. The dominance of phenolic acids, flavan-3-ols, procyanidins, and flavonol glycosides corresponds to chemophenetic peculiarities reported for *Ericaceae* species [5,19,20]. The dominance and consistent accumulation patterns of phenolic compounds are particularly relevant, as these compounds in various studies are associated with antioxidant, anti-inflammatory, and antimicrobial properties [21]. However, no bioactivity assays were performed in the present study; therefore, the observed phenolic profiles indicate phenological stages at which *R. tomentosum* leaves accumulate compound groups that may be relevant for future bioactivity-guided investigations. Recent studies further support the biological relevance of *R. tomentosum* phenolic constituents. Schiavone et al. reported that hydroalcoholic extracts of *R. tomentosum* contained diverse bioactive molecules, including quercetin, catechin, and their derivatives, and demonstrated antioxidant and antimicrobial effects [22]. Similarly, Jasicka-Misiak et al. showed that post-distillation residues of *R. tomentosum* remained a valuable source of phenolic compounds with antioxidant and antimicrobial activity, with solid residues representing a particularly rich phenolic matrix [23]. These findings support the importance of phenolic compounds in the bioactive potential of this species and highlight the relevance of the harvesting period when selecting plant material for phenolic-rich extracts. Flavonoids, phenolic acids, and proanthocyanidins have been widely recognized for their antioxidant activity [24]. Oxidative stress is closely linked with inflammatory processes and carcinogenesis; therefore, phenolic-rich plant extracts suggest potential anti-inflammatory and anticancer activities [21]. In *Rhododendron tomentosum* ssp. *subarcticum*, Black et al. showed that seasonal variation in phenolic constituents was associated with changes in antioxidant and anti-inflammatory activities [17]. Other studies on *Rhododendron* subsect. *Ledum* also emphasize that their flavonoids and phenolic acids contribute to biological activities such as antioxidant, anti-inflammatory, antimicrobial, and antitumor effects [25]. Similar pharmacological relevance of flavonoids, phenolic acids and proanthocyanidins has been reported for other *Ericaceae* plants, including *Vaccinium* species, where phenolic compounds are associated with antioxidant, anti-inflammatory, endothelial-protective, and potential anticancer effects [5,26,27]. The traditional use of *R*. subsection *Ledum* plants as Northern Labrador Tea may therefore provide a culturally relevant source of plant-based antioxidants, particularly in populations with limited fruit and vegetable intake and increased exposure to oxidative stress-inducing substances [28]. The high concentrations of the above-mentioned phenolic origin compounds observed during specific phenological stages may therefore influence the biological activity of plant extracts and should be considered when selecting optimal harvesting periods.

The highest levels of total identified phenolic compounds in *R. tomentosum* leaves were determined during the autumn and winter periods, particularly in samples collected from mid-October to mid-March. This pattern suggests that autumn and winter periods might be associated with the accumulation and maintenance of high levels of phenolic acids, flavan-3-ols, and procyanidin derivatives. A similar dependence of phenolic composition on phenological stage has been reported for *Vaccinium vitis-idaea*, *Arbutus unedo*, *Hakea sericea*, *Acacia melanoxylon* and *Pinus pinaster*, where phenolic profiles varied with phenological stage, with certain compounds favoring autumn and winter periods [6,29,30]. The elevated concentration of phenolic compounds during the autumn and winter period might reflect a phenology-related response under unfavorable environmental conditions [31]. Overwintering evergreen leaves are exposed to a combination of low temperature and light, which might increase the production of reactive oxygen species; therefore, evergreen species rely on photoprotective and antioxidant mechanisms to limit oxidative damage [32]. The proanthocyanidins and other tannins in various studies are associated with resistance to environmental stress, including high radiation, cold stress, and pathogen or herbivore pressure. Their accumulation in overwintering leaves might therefore potentially contribute to tissue protection during the reduced growth period, but leaves remain exposed to environmental stress [33,34]. On the other hand, coniferous swamp forests are commonly characterized by acidic, saturated peat, low nutrient availability and anaerobic conditions [35]. These edaphic peculiarities favor stress-tolerant, slow-growing evergreen species that conserve nutrients through leaves and maintain notable levels of hydroxycinnamic acids, flavan-3-ols and tannins [36]. Therefore, high autumn and winter concentrations of phenolic acids and procyanidins may reflect not only seasonal cold adaptation but also could be the ecological strategy of a stress-tolerant evergreen plant growing in nutrient-limited habitats. The predominance of phenolic acids and procyanidins in autumn and winter samples suggests that this period may be more favorable for collecting *R. tomentosum* leaves rich in total quantified phenolics, especially phenolic acids and proanthocyanidins.

PCA indicates that sample differentiation can be supported by three main metabolic directions (autumn–winter cluster, flowering cluster and fruit development cluster, and transitional early spring cluster) and it is better elucidated than by total phenolic content alone. This agrees with previous findings for *R. tomentosum* ssp. *subarcticum*, where harvest time was significantly affected by catechin, chlorogenic acid, quercetin glycosides and procyanidins [17]. Furthermore, incremental increases in flavonoids during the flowering period have also been reported in other medicinal plants, supporting that reproductive development is accompanied by enhanced flavonol accumulation [37]. Fruit development significantly influenced the allocation of secondary metabolites and was characterized by markedly reduced amounts in leaf samples; this might be explained by the preferential allocation of metabolic resources toward developing reproductive organs [38].

The flowering stage in *R. tomentosum* was characterised by a marked increase in flavonol glycosides, particularly quercitrin, indicating a shift in phenylpropanoid metabolism toward flavonoid accumulation during reproductive development. The flavonoid accumulation is elevated during flowering stages, and this is also highlighted in Ericaceae species, namely *Calluna vulgaris* and *Rhododendron pulchrum* [39,40]. The flavonoids have been reported to contribute to UV screening, antioxidant protection, pollen production and pollination attraction during reproductive stages [41]. The increase in the total flavonoid content during the massive flowering stage was not uniform across all flavonoid subclasses. This pattern was mainly associated with quercetin-type flavonol glycosides, particularly hyperoside, quercitrin, and quercetin-3-O-rhamnoside. On the other hand, the pre-flowering and bud development phases were more clearly associated with increased levels of avicularin, guaijaverin, reynoutrin, spiraeoside, quercetin, and rutin. This pattern might reflect the possible functional role of flavonols in protecting young and actively differentiating tissues, since these compounds are reported to be involved in UV and antioxidant defence, as well as developmental regulation [42,43]. Literature data suggests that flavonoids via the antioxidant mechanisms might promote pollen viability and pollen tube growth and are important during reproductive stages [44]. Overall, the flowering stages may be preferable for plant material collection when flavonol glycosides are the target compounds. The determined correlational interrelationships support the separation of the autumn–winter cluster as a cold-season profile enriched in phenolic acids and procyanidin-type compounds. On the other hand, total flavonoids were not significantly associated with any meteorological factor, suggesting possible linkage to phenological development. The fruit-development cluster, characterized by generally lower concentrations of most phenolic groups, coincided with warmer seasonal conditions.

*Rhododendron* subsect. *Ledum* contains all previous *Ledum* Ruppius ex L. species and now includes 7 species, namely *R. tomentosum*, *R. groenlandicum*, *R. hypoleucum*, *R. diversipilosum*, *R. columbianum*, *R. subulatum* and *R. tolmachevii* [45,46]. Research on *Rhododendron* subsect. *Ledum* is mainly focused on *R. tomentosum* and *R. groenlandicum*, with phytochemical data for other species still scarce [10,47,48,49,50,51,52,53]. Previous studies on *R. tomentosum* have mainly focused on essential oils, volatile constituents, or selected bioactive fractions, whereas detailed information on seasonal changes in non-volatile phenolics remains limited [9,10]. Black et al. investigated Canadian *R. tomentosum* ssp. *subarcticum* during the May–September season and reported significant seasonal variation in catechin, chlorogenic acid, quercetin glycosides, and procyanidins B1-B3. Overall, their study identified two maxima in the total number of identified compounds during the flowering and late September periods [17]. Our results correspond to the qualitative profiles of the determined quercetin derivatives, chlorogenic acid, catechin, and proanthocyanidins, although, by covering a full annual phenological cycle, our results extend the findings and further highlight the importance of collection time. In comparison with *R. groenlandicum*, data on the *R. tomentosum* phenolic profiles highlight the presence of the taxifolin derivatives and lower amounts of catechin, epicatechin and proanthocyanidins [19]. Black et al. reported the (+)-catechin predominance in *R. tomentosum*, while Saleem et al. identified taxifolin and taxifolin glycoside as prevailing markers of this species. These differences suggest that, although both species share flavan-3-ols and flavonol derivatives, their phytochemical profiles are different and might be significantly impacted by environmental and seasonal conditions [9,10,17,18,47,48,49,50,51,52,53]. Therefore, the present results fill the gap in earlier studies by showing that the phenolic composition of naturally growing *R. tomentosum* leaves is not only diverse phytogeographically but also strongly influenced by phenological stage.

## 4. Materials and Methods

### 4.1. Chemicals

All solvents used for chromatographic analysis were of HPLC grade. Acetonitrile was purchased from Honeywell Riedel-de Haën (Berlin, Germany), and trifluoroacetic acid (TFA) was obtained from Merck Schuchardt (Hohenbrunn, Germany). Ethanol (96%, *v*/*v*) was obtained from AB Vilniaus Degtinė (Vilnius, Lithuania). Reference standards of neochlorogenic acid, chlorogenic acid, catechin, fraxetin, epicatechin, taxifolin, procyanidin A1, rutin, hyperoside, isoquercitrin, reynoutrin, guaijaverin, avicularin, quercitrin, spiraeoside, and quercetin were purchased from Sigma-Aldrich (Buchs, Switzerland). Ultrapure water was produced using a Milli-Q water purification system (Millipore, Bedford, MA, USA).

### 4.2. Plant Materials and Growing Conditions

Leaves of *Rhododendron tomentosum* Harmaja were collected from a natural population located in the Dubrava (Kaunas district, Lithuania; 54.848069° N, 24.078781° E), representing a coniferous swamp forest habitat characterised by acidic peat soils, high moisture content, and nutrient-poor conditions. Sampling was conducted over one complete annual vegetation cycle, from October 2023 to October 2024. Twenty sampling events (DT1–DT20) were conducted to cover all major phenological stages, including autumn vegetative growth, winter dormancy, early-spring vegetation, bud formation, flowering, fruit development, and late-season vegetation. The identity of the raw material was established by Lina Raudonė, professor of the Department of Pharmacognosy, Faculty of Pharmacy, Lithuanian University of Health Sciences, Kaunas, Lithuania. A voucher specimen was deposited at the Herbarium of Pharmacognosy department Faculty of Pharmacy, Lithuanian University of Health Sciences, under number RT2023. Detailed information on sampling dates, phenological stages, and meteorological conditions is provided in Table 5. Particular attention was given to the transition from dormancy to active growth. Therefore, two distinct micro-phenological stages were sampled on the same date (18 March 2024): DT8, representing plants with brown buds, and DT9, representing plants with green buds, thereby allowing assessment of phytochemical variation during early bud development.

During each sampling event, three independent biological replicates were collected. Each biological replicate consisted of leaves pooled from ten randomly selected individual plants growing within the same habitat. Plant material was air-dried at room temperature, ground to a homogeneous powder, and stored until further extraction and analysis. Meteorological data, including air temperature, precipitation, cloudiness, and relative humidity, were obtained for each sampling event and are summarised in Table 5.

### 4.3. Preparation of Extracts

Each biological replicate was extracted and analysed independently. About 0.2 g of fine powder of dried leaves was extracted with 10 mL 70% ethanol in water for 15 min in Elmasonic P ultrasonic bath (Elma Schmidbauer GmbH, Singen, Germany). After that, samples were put in a centrifuge at 4000× *g* for 10 min in a Biofuge Stratos centrifuge (Thermo Electron Corporation, Hanau, Germany), and the supernatants were filtered through 0.22 μm pore size membrane filters (Carl Roth GmbH, Karlsruhe, Germany) and transferred to the dark glass vials.

### 4.4. HPLC-PDA Conditions

**Figure 3:**
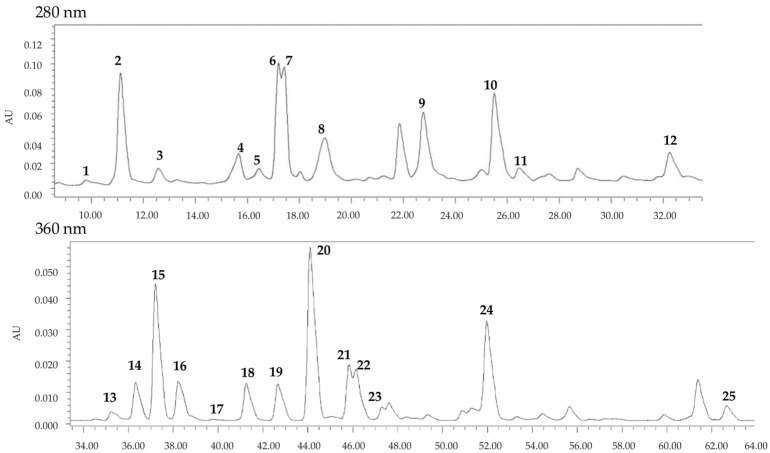
Representative HPLC–PDA chromatograms of *Rhododendron tomentosum* leaf extract recorded at 280 nm and 360 nm. Detection at 280 nm was selected for the visualization of phenolic acids, coumarin derivatives, flavan-3-ols and procyanidin-type compounds, whereas detection at 360 nm was used for the visualization of the main flavonoid compounds. Peak identification: 1—gallocatechin; 2—neochlorogenic acid; 3—esculetin-O-hexoside; 4—procyanidin derivative 1; 5—procyanidin derivative 2; 6—chlorogenic acid; 7—catechin; 8—fraxetin-O-hexoside; 9—epicatechin; 10—taxifolin-O-hexoside; 11—gallocatechin gallate; 12—procyanidin A1; 13—rutin; 14—quercetin-O-diglycoside; 15—hyperoside; 16—isoquercitrin; 17—procyanidin A; 18—reynoutrin; 19—guaijaverin; 20—avicularin; 21—quercitrin; 22—quercetin-3-O-rhamnoside isomer; 23—spiraeoside; 24—quercetin-type flavonol glycoside derivative; 25—quercetin.

Phenolic compounds were analysed using a high-performance liquid chromatography system equipped with a photodiode array detector (HPLC–PDA) (Waters Corporation (Milford, MA, USA) e2695 separation module coupled with a 2998 PDA detector). Chromatographic separation was performed on a reversed-phase C18 column (Ace Excel 3 super, 250 × 4.6 mm (Advanced Chromatography Technologies Ltd., Aberdeen, UK), maintained at 35 °C. The mobile phase consisted of solvent A (water with 0.1% Trifluoroacetic acid) and solvent B (100% acetonitrile). Gradient elution was applied as follows: 1–65 min—A 90%; B 10%, 65–80 min—A 70%; B 30%, 80 min—A 30%; B 70%, 83–84 min—A 10%; B 90%, 85–90 min A90%; B10%. The flow rate was 0.6 mL/min, and the injection volume was 10 µL. Detection was carried out over the range of 200–400 nm, with chromatograms recorded at 360 nm, which is characteristic for flavonoid compounds. Additional wavelengths (e.g., 280 nm and 320 nm) were used to support the identification of flavan-3-ols and phenolic acids. Identification of compounds was based on retention times, UV–Vis spectral characteristics (λ max), and comparison with reference standards and literature data. Quantification was performed using external calibration curves, and results were expressed as µg/g dry weight (DW). Compounds without available reference standards were tentatively identified by comparing their UV-Vis spectral characteristics, chromatographic behavior, and published data reported for *Rhododendron* species and other members of the *Ericaceae* family, Figure 3.

The analytical method was validated in terms of linearity, sensitivity, precision, and accuracy. Calibration curves were constructed using authentic reference standards at six concentration levels within the range of 1.56–100 µg/mL. All calibration curves demonstrated excellent linearity, with coefficients of determination (R^2^) ranging from 0.999673 to 0.999998. Method sensitivity was evaluated by determining the limits of detection (LOD) and quantification (LOQ), while precision was assessed through intra-day (*n* = 6) and inter-day (*n* = 3) repeatability experiments. Accuracy was evaluated through recovery studies. The validation parameters obtained for all quantified compounds are summarized in Table 6.

### 4.5. Spectrophotometric Determination of Total Phenolic Groups

Spectrophotometric assays were performed to complement the HPLC–PDA characterization of individual phenolic compounds and to obtain integrated estimates of the major phenolic groups in *R. tomentosum* leaf extracts. Total phenolic content (TPC), total flavonoid content (TFC), total hydroxycinnamic acid content (HCA), and total proanthocyanidin content (TPAC) were determined using gallic acid, rutin, chlorogenic acid, and (−)-epicatechin calibration curves, respectively. Absorbance measurements were carried out using a Dynamica HALO DB-20 UV–Vis spectrophotometer (Dynamica GmbH, Dietikon, Switzerland) at 765 nm (TPC), 407 nm (TFC), 525 nm (HCA), and 640 nm (TPAC). Extracts were diluted when necessary to ensure that absorbance values remained within the linear range of the corresponding calibration curves. Results were expressed as mg gallic acid equivalents (GAE), rutin equivalents (RE), chlorogenic acid equivalents (CRE), and epicatechin equivalents (EE) per g dry weight (DW). All measurements were performed in triplicate.

### 4.6. Statistical Analysis

All analyses were performed in triplicate, and the results were expressed as mean ± standard deviation (SD). Statistical differences among sampling periods were evaluated using one-way analysis of variance (ANOVA), followed by Tukey’s post hoc test for multiple comparisons. The results of Tukey’s HSD post hoc test are presented in Table 1, Table 2 and Table 3 as lowercase grouping letters to indicate significant differences among sampling periods. Differences were considered statistically significant at *p* < 0.05. Relationships between meteorological parameters and phenolic compound accumulation were assessed using Spearman’s rank correlation analysis. Correlations were considered statistically significant at *p* < 0.05. To evaluate relationships among phenolic compounds and to identify patterns associated with different phenological stages, principal component analysis (PCA) was performed using the standardized concentration data. The suitability of the dataset for PCA was assessed using the Kaiser–Meyer–Olkin (KMO) measure of sampling adequacy and Bartlett’s test of sphericity. Principal components with eigenvalues greater than 1.0 were retained, and varimax rotation was applied to facilitate interpretation of the loading structure. All statistical analyses were performed using IBM SPSS Statistics version 31.0 (IBM Corp., Armonk, NY, USA), while graphical representation and data processing were carried out using Microsoft Excel version 16.100.1 (Microsoft Corporation, Redmond, WA, USA).

## 5. Conclusions

This study demonstrated that the phenological stage significantly influences the quantitative and qualitative phenolic profile of *Rhododendron tomentosum*. A full annual sampling approach revealed three main phenological chemical directions: an autumn–winter dormancy profile (often omitted in seasonal phytochemical studies) characterized by the highest total identified phenolic content and the predominance of phenolic acids, flavan-3-ols and procyanidin-type compounds, a flowering-stage profile associated with increased accumulation of quercetin glycosides, and a fruit-development profile characterized by reduced levels of most quantified leaf phenolics.

The observed seasonal patterns suggest that the optimal harvesting period should be selected according to the target compound group. Further studies involving multiple populations, different climatic zones, repeated annual cycles, and complementary physiological and bioactivity assays are needed to validate the broader applicability and functional relevance of the observed phenological-phytochemical patterns.

## Figures and Tables

**Table 1:** Seasonal variation in phenolic acids, flavan-3-ols, coumarin derivatives, and procyanidin-type compounds in *R. tomentosum* leaves during different phenological stages.

Code	Phenological Stage	Galocatechin	Neochlorogenic Acid	Esculetin-O-Hexoside	Procyanidin Derivative 1	Procyanidin Derivative 2	Chlorogenic Acid	Catechin	Fraxetin-O-Hexoside	Epicatechin
DT1	autumn vegetative stage	469.6 ± 24.9 ^a^	7455.2 ± 335 ^a^	1207.2 ± 153.1 ^ab^	8323.4 ± 184.5 ^a^	3867.8 ± 12.6 ^abc^	8268.6 ± 219.0 ^e^	3785.3 ± 98.0 ^j^	4018.0 ± 71.8 ^efg^	3402.7 ± 531.1 ^efg^
DT2	autumn vegetative stage	238.1 ± 9.5 ^bc^	7000.4 ± 46.4 ^a^	902.1 ± 51.5 ^cd^	8226.2 ± 275.7 ^a^	3790.2 ± 185.1 ^bc^	12,596.5 ± 332.0 ^b^	8543.0 ± 228.0 ^cd^	4443.8 ± 162.8 ^de^	3146.2 ± 79.7 ^efg^
DT3	winter dormancy	127.2 ± 2 ^ghi^	7839.3 ± 392.5 ^a^	1301.9 ± 87.7 ^ab^	7938.7 ± 542.7 ^a^	4169.8 ± 209.3 ^ab^	16,339.2 ± 348.0 ^a^	8252.2 ± 220.0 ^d^	5163.9 ± 254.7 ^bc^	5245.3 ± 511.3 ^ab^
DT4	winter dormancy	180.1 ± 1.3 ^cdefg^	8744.3 ± 465.3 ^a^	1257.3 ± 91.5 ^ab^	7027.8 ± 363.5 ^bc^	3630.2 ± 121.0 ^cd^	5812.2 ± 156.0 ^gh^	8865.6 ± 237.0 ^c^	5123.7 ± 202.3 ^bc^	5708.8 ± 563.6 ^a^
DT5	winter dormancy	232.1 ± 5.3 ^bcd^	6445.6 ± 294.5 ^a^	1189.0 ± 109.3 ^ab^	8164.4 ± 551.6 ^a^	4190.6 ± 169.5 ^a^	9373.2 ± 245.0 ^d^	9574.7 ± 255.0 ^b^	5137.2 ± 307.9 ^bc^	4679.1 ± 152.0 ^bc^
DT6	winter dormancy	268.2 ± 68.5 ^b^	6326.7 ± 275.5 ^a^	1102.9 ± 17.9 ^bc^	6873.7 ± 192.0 ^cd^	3614.5 ± 100.1 ^cd^	10,297.9 ± 274.0 ^c^	6015.1 ± 162.0 ^g^	5730.2 ± 164.0 ^a^	5127.3 ± 344.5 ^ab^
DT7	winter dormancy	178.9 ± 4.1 ^cdefg^	6509.2 ± 103 ^a^	805.0 ± 17.8 ^de^	6279.3 ± 269.1 ^cd^	3287.3 ± 169.4 ^de^	12,259.8 ± 321.0 ^b^	7660.5 ± 207.0 ^e^	4350.2 ± 136.4 ^de^	5061.9 ± 149.2 ^ab^
DT8	early spring vegetation	211.4 ± 4.9 ^bcde^	6638.7 ± 80.3 ^a^	1200.2 ± 81.0 ^ab^	6673.2 ± 192.4 ^cd^	3763.0 ± 84.5 ^c^	5216.1 ± 142.0 ^hi^	6689.9 ± 181.0 ^f^	5719.9 ± 122.5 ^a^	5662.6 ± 82.7 ^a^
DT9	early spring vegetation	189.7 ± 2.7 ^cdef^	5748.4 ± 93.5 ^a^	1342.0 ± 69.5 ^a^	7856.5 ± 149.1 ^ab^	3719.6 ± 47.8 ^c^	6912.3 ± 185.0 ^f^	8833.9 ± 238.0 ^c^	4393.3 ± 106.8 ^de^	4458.8 ± 111.0 ^bcd^
DT10	early spring vegetation	154.1 ± 27.4 ^efgh^	5510.2 ± 52.3 ^a^	910.4 ± 33.6 ^cd^	5078.3 ± 253.7 ^e^	2738.9 ± 33.2 ^fg^	4830.7 ± 131.0 ^ij^	4385.3 ± 118.0 ^i^	4451.1 ± 59.6 ^de^	2864.6 ± 232.7 ^fg^
DT11	bud formation	125 ± 34.4 ^ghi^	4152.4 ± 42 ^a^	752.7 ± 12.0 ^def^	4556.9 ± 232.5 ^e^	2331.6 ± 76.8 ^h^	3577.2 ± 92.0 ^lm^	2050.9 ± 56.0 ^k^	3997.9 ± 83.2 ^efg^	1970.2 ± 232.2 ^hi^
DT12	early flowering	104.6 ± 2 ^hij^	4884 ± 203.4 ^a^	463.6 ± 29.8 ^g^	3653.2 ± 294.0 ^fg^	2027.8 ± 145.6 ^hij^	3108.8 ± 81.0 ^m^	1708.3 ± 47.0 ^kl^	3764.0 ± 216.2 ^fgh^	2646.2 ± 78.2 ^gh^
DT13	flowering	61.5 ± 5.9 ^j^	3007.3 ± 50.8 ^a^	502.0 ± 17.8 ^g^	4362.3 ± 142.6 ^ef^	2222.8 ± 120.2 ^hi^	9449.8 ± 251.0 ^d^	3377.3 ± 92.0 ^j^	4085.3 ± 305.0 ^ef^	1997.2 ± 493.5 ^hi^
DT14	flowering	147 ± 3 ^fgh^	2575 ± 75.4 ^a^	606.1 ± 22.9 ^efg^	4352.5 ± 240.1 ^ef^	2319.4 ± 83.3 ^h^	4305.2 ± 117.0 ^jk^	1696.3 ± 47.0 ^kl^	3556.3 ± 164.2 ^gh^	1309.3 ± 185.4 ^ij^
DT15	fruit development	147.2 ± 4.4 ^fgh^	1228.2 ± 3.1 ^a^	551.3 ± 14.4 ^fg^	3670.3 ± 12.1 ^fg^	1911.4 ± 43.4 ^ij^	3746.6 ± 98.0 ^kl^	1833.9 ± 51.0 ^kl^	2426.3 ± 16.8 ^i^	818.7 ± 1.3 ^j^
DT16	fruit development	75.2 ± 6 ^ij^	1616.6 ± 18.4 ^a^	499.3 ± 13.6 ^g^	2294.9 ± 34.8 ^h^	1218.2 ± 18.0 ^k^	4858.7 ± 128.0 ^ij^	1469.8 ± 41.0 ^l^	3385.7 ± 35.1 ^h^	675.9 ± 106.1 ^j^
DT17	fruit development	55.9 ± 1.1 ^j^	1814.6 ± 70.6 ^a^	839.0 ± 3.8 ^d^	4630.9 ± 156.7 ^e^	2376.5 ± 6.4 ^gh^	3054.9 ± 79.0 ^m^	3706.8 ± 96.0 ^j^	2759.5 ± 14.8 ^i^	1206.3 ± 13.1 ^ij^
DT18	fruit development	175.4 ± 6.1 ^defg^	3096.1 ± 29.1 ^a^	939.0 ± 55.4 ^cd^	4864.5 ± 440.3 ^e^	2248.1 ± 231.6 ^hi^	2997.6 ± 76.0 ^m^	4895.4 ± 132.0 ^h^	4316.5 ± 169.0 ^de^	3613.9 ± 236.6 ^def^
DT19	autumn vegetative stage	142.2 ± 5.8 ^fgh^	2498.5 ± 204.2 ^a^	628.3 ± 25.9 ^efg^	3342.8 ± 278.2 ^g^	1680.5 ± 118.1 ^j^	3008.2 ± 82.0 ^m^	1778.7 ± 49.0 ^kl^	4676.1 ± 91.0 ^cd^	1893.2 ± 191.9 ^hi^
DT20	autumn vegetative stage	479.8 ± 15.6 ^a^	6782.6 ± 118.4 ^a^	1108.9 ± 128.6 ^bc^	6112.6 ± 181.4 ^d^	2931.7 ± 115.4 ^ef^	6029.1 ± 164.0 ^g^	10,839.4 ± 290.0 ^a^	5250.6 ± 127.5 ^ab^	3916.0 ± 58.8 ^cde^
	F	101.025	1.011	58.522	128.828	161.995	1124.658	1096.438	88.558	107.549
	Anova *p*	<0.001	0.471	<0.001	<0.001	<0.001	<0.001	<0.001	<0.001	<0.001

Values are expressed as mean ± SD (*n* = 3). Different lowercase letters within the same column indicate statistically significant differences between sampling periods according to Tukey’s HSD test (*p* < 0.05). Values sharing at least one common letter are not significantly different. Letters should be interpreted separately for each compound.

**Table 2:** Seasonal variation in flavonoid glycosides and procyanidin A1 in *R. tomentosum* leaves during different phenological stages.

Code	Phenological Stage	Taxifolin-O-Hexoside	Gallocatechin Gallate	Procyanidin A1	Rutin	Quercetin-O-Diglycoside	Hyperoside	Isoquercitrin	Procyanidin A	Reynoutrin
DT1	autumn vegetative stage	993.6 ± 18.3 ^fg^	1291.3 ± 144.4 ^ef^	5809.1 ± 202.0 ^a^	259.4 ± 0.5 ^abc^	239.1 ± 15.1 ^ab^	2792.2 ± 106.4 ^a^	576.1 ± 25.6 ^cde^	2150.5 ± 104.7 ^hi^	516.3 ± 20.1 ^fgh^
DT2	autumn vegetative stage	1486.6 ± 18.5 ^bc^	1483.9 ± 44.6 ^d^	5721.9 ± 178.7 ^ab^	61.8 ± 0.1 ^g^	164.7 ± 6.7 ^abc^	2680.2 ± 85.5 ^ab^	582.2 ± 21.1 ^cde^	2865.1 ± 96.3 ^fg^	665.5 ± 12.6 ^b^
DT3	winter dormancy	1537.2 ± 100.7 ^bc^	992.7 ± 37.7 ^g^	5394.3 ± 272.0 ^abc^	123.2 ± 4.0 ^efg^	216.7 ± 52.0 ^abc^	2292.4 ± 162.5 ^d^	806.4 ± 193.6 ^a^	3921.5 ± 184.6 ^abc^	621.9 ± 28.8 ^bcd^
DT4	winter dormancy	2049.7 ± 151.8 ^a^	1221.0 ± 124.5 ^ef^	4765.2 ± 157.9 ^def^	99.4 ± 8.0 ^fg^	199.5 ± 49.9 ^abc^	2619.6 ± 123.5 ^abc^	505.4 ± 13.8 ^cdef^	3545.8 ± 201.6 ^cd^	503.6 ± 30.3 ^fghi^
DT5	winter dormancy	1619.9 ± 121.8 ^b^	1964.1 ± 94.7 ^ab^	5262.1 ± 278.8 ^bcd^	111.0 ± 22.0 ^efg^	203.0 ± 38.3 ^abc^	2177.8 ± 113.8 ^de^	429.8 ± 8.7 ^ef^	3046.7 ± 245.9 ^ef^	492.8 ± 40.4 ^fghij^
DT6	winter dormancy	1078.8 ± 66.2 ^efg^	1370.5 ± 11.7 ^def^	4819.6 ± 129.3 ^de^	234.6 ± 51.1 ^abcd^	221.7 ± 47.1 ^ab^	2274.7 ± 139.2 ^d^	681.3 ± 154.4 ^abc^	4056.4 ± 130.1 ^a^	589.9 ± 34.0 ^cde^
DT7	winter dormancy	1245.6 ± 46.2 ^de^	960.0 ± 16.0 ^gh^	5371.4 ± 217.0 ^abc^	328.4 ± 23.4 ^a^	187.1 ± 15.8 ^abc^	2360.7 ± 87.6 ^cd^	633.9 ± 40.7 ^abcd^	2510.7 ± 71.6 ^gh^	673.4 ± 19.2 ^b^
DT8	early spring vegetation	1494.5 ± 39.9 ^bc^	1299.4 ± 14.9 ^ef^	5182.6 ± 179.9 ^cd^	283.6 ± 11.4 ^ab^	207.4 ± 7.7 ^abc^	2422.5 ± 27.4 ^bcd^	462.7 ± 35.4 ^def^	3960.7 ± 52.4 ^ab^	620.5 ± 8.8 ^bcd^
DT9	early spring vegetation	1138.7 ± 26.2 ^defg^	899.3 ± 37.0 ^gh^	4982.7 ± 103.1 ^cde^	67.2 ± 1.9 ^g^	183.4 ± 6.3 ^abc^	1878.0 ± 36.7 ^f^	381.3 ± 9.4 ^f^	2175.5 ± 34.4 ^hi^	765.3 ± 19.3 ^a^
DT10	early spring vegetation	1347.3 ± 155.9 ^cd^	1322.8 ± 32.2 ^def^	4634.9 ± 341.7 ^efg^	176.7 ± 19.0 ^cdef^	124.3 ± 36.2 ^bc^	1899.2 ± 40.9 ^f^	368.4 ± 19.7 ^f^	3314.3 ± 106.2 ^de^	477.9 ± 18.9 ^ghij^
DT11	bud formation	1144.2 ± 43.9 ^def^	968.2 ± 62.5 ^gh^	3965.9 ± 96.2 ^hi^	155.1 ± 7.7 ^defg^	121.0 ± 36.1 ^bc^	1511.5 ± 36.4 ^g^	322.9 ± 22.7 ^f^	3426.2 ± 167.6 ^de^	431.5 ± 12.8 ^jk^
DT12	early flowering	1586.0 ± 80.0 ^b^	1379.7 ± 18.9 ^de^	4290.5 ± 207.8 ^fgh^	127.7 ± 28.0 ^efg^	120.2 ± 11.4 ^bc^	2796.4 ± 62.4 ^a^	629.5 ± 23.0 ^abcd^	3410.4 ± 202.7 ^de^	451.6 ± 21.6 ^hijk^
DT13	flowering	585.9 ± 20.8 ^h^	830.2 ± 80.5 ^gh^	3513.8 ± 68.3 ^ij^	117.4 ± 2.6 ^efg^	119.8 ± 1.1 ^bc^	2661.3 ± 41.8 ^ab^	632.5 ± 11.2 ^abcd^	2803.1 ± 158.7 ^fg^	638.2 ± 10.0 ^bc^
DT14	flowering	665.5 ± 36.8 ^h^	806.8 ± 31.0 ^h^	2785.1 ± 116.4 ^k^	140.5 ± 85.6 ^defg^	164.6 ± 14.3 ^abc^	2238.9 ± 98.4 ^d^	598.1 ± 29.5 ^bcde^	1972.4 ± 96.4 ^i^	632.0 ± 26.8 ^bc^
DT15	fruit development	619.0 ± 6.5 ^h^	1691.0 ± 2.9 ^c^	3036.4 ± 30.8 ^jk^	253.8 ± 25.7 ^abc^	113.6 ± 6.9 ^bc^	1146.5 ± 13.5 ^h^	370.1 ± 16.3 ^f^	1209.4 ± 3.9 ^j^	436.9 ± 1.6 ^ijk^
DT16	fruit development	487.3 ± 26.8 ^h^	971.0 ± 28.7 ^gh^	2647.7 ± 17.3 ^k^	117.4 ± 11.6 ^efg^	129.3 ± 14.3 ^bc^	1951.3 ± 39.6 ^ef^	780.3 ± 34.9 ^ab^	2476.7 ± 66.2 ^gh^	785.2 ± 14.1 ^a^
DT17	fruit development	1616.1 ± 82.1 ^b^	1193.9 ± 18.4 ^f^	3829.2 ± 13.9 ^hi^	201.9 ± 10.2 ^bcde^	173.1 ± 29.7 ^abc^	1899.2 ± 40.8 ^f^	593.9 ± 24.3 ^cde^	1973.0 ± 18.3 ^i^	538.1 ± 24.2 ^efg^
DT18	fruit development	1157.2 ± 106.2 ^def^	933.3 ± 21.2 ^gh^	4180.1 ± 80.1 ^gh^	152.8 ± 81.8 ^defg^	120.5 ± 38.2 ^bc^	1946.1 ± 97.1 ^ef^	350.4 ± 32.2 ^f^	3561.8 ± 72.7 ^cd^	463.9 ± 29.9 ^hijk^
DT19	autumn vegetative stage	906.7 ± 64.9 ^g^	1805.9 ± 43.6 ^bc^	3639.8 ± 136.3 ^i^	135.1 ± 1.1 ^efg^	278.7 ± 138.7 ^a^	1390.6 ± 74.2 ^gh^	376.8 ± 9.0 ^f^	3592.2 ± 110.8 ^bcd^	559.3 ± 10.4 ^def^
DT20	autumn vegetative stage	1553.9 ± 21.7 ^bc^	2018.3 ± 32.9 ^a^	5128.5 ± 81.3 ^cde^	136.7 ± 1.1 ^efg^	89.8 ± 2.6 ^c^	2556.8 ± 33.2 ^abc^	467.2 ± 6.6 ^def^	3327.4 ± 56.0 ^de^	401.4 ± 2.9 ^k^
	F	90.531	120.569	96.846	16.049	4.467	94.227	17.627	114.563	76.576
	Anova *p*	<0.001	<0.001	<0.001	<0.001	<0.001	<0.001	<0.001	<0.001	<0.001

Values are expressed as mean ± SD (*n* = 3). Different lowercase letters within the same column indicate statistically significant differences between sampling periods according to Tukey’s HSD test (*p* < 0.05). Values sharing at least one common letter are not significantly different. Letters should be interpreted separately for each compound.

**Table 3:** Seasonal variation in quercetin derivatives and flavonol glycosides in *R. tomentosum* leaves during different phenological stages.

Code	Phenological Stage	Guaijaverin	Avicularin	Quercitrin	Quercetin-3-O-Rhamnoside Isomer	Spiraeoside	Quercetin Type Flavonol Glycoside Derivative	Quercetin
DT1	autumn vegetative stage	675.6 ± 26.9 ^a^	5327.0 ± 198.5 ^cde^	576.2 ± 38.7 ^i^	166.8 ± 1.4 ^hi^	106.1 ± 11.9 ^bc^	607.2 ± 17.3 ^bcd^	112.4 ± 1.7 ^cd^
DT2	autumn vegetative stage	662.8 ± 16.2 ^a^	5809.3 ± 143.2 ^abc^	1035.1 ± 18.8 ^fghi^	240.5 ± 0.4 ^de^	48.8 ± 0.8 ^gh^	565.9 ± 15.5 ^def^	110.7 ± 1.3 ^cde^
DT3	winter dormancy	667.8 ± 32.2 ^a^	6098.2 ± 296.2 ^ab^	972.2 ± 80.2 ^ghi^	181.4 ± 10.1 ^ghi^	48.7 ± 0.9 ^gh^	688.3 ± 34.1 ^a^	97.4 ± 2.6 ^ijk^
DT4	winter dormancy	581.5 ± 31.8 ^a^	4860.3 ± 254.2 ^efgh^	921.8 ± 46.0 ^ghi^	192.4 ± 6.3 ^fgh^	58.2 ± 0.5 ^fgh^	538.4 ± 18.6 ^fg^	106.7 ± 2.3 ^defg^
DT5	winter dormancy	470.9 ± 35.5 ^a^	4566.4 ± 276.4 ^ghi^	1012.2 ± 71.6 ^fghi^	165.6 ± 6.0 ^hi^	87.5 ± 10.2 ^cde^	453.5 ± 25.4 ^h^	104.7 ± 4.1 ^efgh^
DT6	winter dormancy	654.9 ± 29.5 ^a^	5824.9 ± 257.3 ^abc^	821.4 ± 32.2 ^hi^	156.5 ± 4.9 ^i^	71.4 ± 7.6 ^efg^	627.9 ± 15.9 ^bc^	104.1 ± 1.0 ^fgh^
DT7	winter dormancy	629.4 ± 20.9 ^a^	5900.2 ± 178.6 ^ab^	587.4 ± 9.1 ^i^	263.4 ± 4.7 ^cd^	59.2 ± 6.5 ^fgh^	635.5 ± 29.6 ^ab^	107.5 ± 2.5 ^defg^
DT8	early spring vegetation	617 ± 9.1 ^a^	5597.3 ± 75.3 ^bcd^	1452.4 ± 337.5 ^efg^	241.5 ± 34.2 ^de^	124.5 ± 1.5 ^b^	564.4 ± 9.8 ^def^	120.6 ± 1.6 ^b^
DT9	early spring vegetation	686.9 ± 15 ^a^	6177.5 ± 116.2 ^a^	1906.6 ± 91.9 ^e^	236.8 ± 3.9 ^de^	179.6 ± 1.6 ^a^	576.9 ± 12.6 ^cdef^	143.5 ± 2.4 ^a^
DT10	early spring vegetation	540.5 ± 14.9 ^a^	4705.7 ± 120.6 ^fghi^	1948.6 ± 312.9 ^e^	221.0 ± 17.8 ^ef^	60.1 ± 5.4 ^fgh^	549.3 ± 24.5 ^ef^	106.5 ± 0.7 ^defg^
DT11	bud formation	476.5 ± 12.7 ^a^	4326.5 ± 88.7 ^i^	1259.1 ± 13.7 ^fgh^	209.7 ± 9.3 ^efg^	70.6 ± 17.3 ^efg^	480.2 ± 15.6 ^h^	109.7 ± 2.4 ^cdef^
DT12	early flowering	625.3 ± 23.5 ^a^	4533.0 ± 203.7 ^ghi^	1583.0 ± 60.4 ^ef^	712.4 ± 15.2 ^a^	52.2 ± 21.2 ^gh^	468.1 ± 19.8 ^h^	111.2 ± 4.4 ^cde^
DT13	flowering	651.9 ± 15.5 ^a^	4979.9 ± 96.4 ^efg^	19,517.1 ± 171.0 ^a^	701.2 ± 12.3 ^a^	58.6 ± 3.9 ^fgh^	437.6 ± 10.6 ^h^	107.2 ± 2.2 ^defg^
DT14	flowering	596 ± 23.8 ^a^	4301.1 ± 114.3 ^ij^	15,431.9 ± 604.6 ^b^	664.4 ± 9.3 ^a^	69.2 ± 0.8 ^efgh^	490.4 ± 10.7 ^gh^	98.5 ± 1.8 ^hij^
DT15	fruit development	507.1 ± 6.3 ^a^	3682.2 ± 16.9 ^k^	4309.3 ± 40.5 ^d^	631.8 ± 14.8 ^a^	77.6 ± 3.5 ^ef^	380.9 ± 3.1 ^i^	87.3 ± 0.5 ^l^
DT16	fruit development	666.8 ± 10.7 ^a^	5120.8 ± 115.2 ^def^	6784.4 ± 107.9 ^c^	569.1 ± 17.3 ^a^	86.2 ± 1.5 ^cde^	602.6 ± 8.6 ^bcde^	91.2 ± 0.3 ^kl^
DT17	fruit development	562.2 ± 20.1 ^a^	4509.7 ± 149.9 ^ghi^	4646.8 ± 218.9 ^d^	561.1 ± 14.9 ^a^	86.8 ± 1.5 ^cde^	487.6 ± 10.8 ^gh^	102.6 ± 0.3 ^ghi^
DT18	fruit development	563.4 ± 34.5 ^a^	4627.1 ± 218.8 ^fghi^	669.8 ± 18.5 ^i^	321.0 ± 15.2 ^b^	103.6 ± 10.1 ^bcd^	483.7 ± 24.4 ^gh^	91.9 ± 2.0 ^jkl^
DT19	autumn vegetative stage	543.6 ± 7.2 ^a^	4435.5 ± 106.8 ^hi^	828.0 ± 32.0 ^hi^	228.5 ± 1.3 ^e^	80.3 ± 0.8 ^def^	490.5 ± 8.0 ^gh^	107.7 ± 1.4 ^defg^
DT20	autumn vegetative stage	518.3 ± 20.2 ^a^	3793.2 ± 40.6 ^jk^	754.2 ± 7.2 ^hi^	289.8 ± 5.1 ^bc^	45.6 ± 1.1 ^h^	600.8 ± 9.1 ^bcde^	114.7 ± 1.3 ^bc^
	F	1.002	56.437	2267.406	683.379	47.826	56.712	94.147
	Anova *p*	0.479	<0.001	<0.001	<0.001	<0.001	<0.001	<0.001

Values are expressed as mean ± SD (*n* = 3). Different lowercase letters within the same column indicate statistically significant differences between sampling periods according to Tukey’s HSD test (*p* < 0.05). Values sharing at least one common letter are not significantly different. Letters should be interpreted separately for each compound.

**Table 4:** Seasonal variation in spectrophotometrically determined phenolic groups in *R. tomentosum* leaves collected during different phenological stages.

Sample	TPC (mg GAE/g DW)	TFC (mg RE/g DW)	HCA (mg CRE/g DW)	TPAC (mg EE/g DW)
DT1	31.14 ± 0.96	19.71 ± 0.58	114.80 ± 2.24	1.44 ± 0.07
DT2	44.90 ± 1.35	28.50 ± 0.85	118.00 ± 3.54	2.25 ± 0.11
DT3	53.60 ± 1.61	32.40 ± 0.97	165.48 ± 4.93	2.99 ± 0.15
DT4	41.79 ± 1.24	27.50 ± 0.82	128.50 ± 3.85	2.64 ± 0.13
DT5	53.80 ± 1.61	25.33 ± 0.82	154.40 ± 4.63	3.23 ± 0.16
DT6	32.11 ± 0.98	16.80 ± 0.50	104.60 ± 3.14	1.60 ± 0.08
DT7	42.80 ± 1.28	27.37 ± 0.86	129.08 ± 3.87	2.23 ± 0.11
DT8	37.89 ± 1.16	19.10 ± 0.57	129.48 ± 3.86	2.37 ± 0.12
DT9	34.20 ± 1.03	19.17 ± 0.58	112.70 ± 3.38	1.94 ± 0.10
DT10	31.28 ± 0.97	15.34 ± 0.45	93.67 ± 2.84	1.70 ± 0.09
DT11	27.40 ± 0.82	15.70 ± 0.47	87.83 ± 1.45	1.41 ± 0.07
DT12	26.29 ± 0.77	12.21 ± 0.41	90.80 ± 2.72	1.29 ± 0.06
DT13	40.90 ± 1.23	18.30 ± 0.55	105.03 ± 3.19	1.87 ± 0.09
DT14	26.10 ± 0.80	11.40 ± 0.34	82.50 ± 2.48	1.15 ± 0.06
DT15	32.49 ± 0.99	18.49 ± 0.55	89.06 ± 2.74	1.26 ± 0.06
DT16	32.60 ± 0.98	19.80 ± 0.59	88.80 ± 2.66	1.21 ± 0.06
DT17	36.03 ± 1.09	19.07 ± 0.57	97.10 ± 2.91	1.71 ± 0.09
DT18	39.49 ± 1.18	11.82 ± 0.35	103.20 ± 3.10	2.08 ± 0.10
DT19	36.70 ± 1.10	15.63 ± 0.47	86.60 ± 2.60	1.53 ± 0.08
DT20	43.65 ± 1.38	18.81 ± 0.56	124.59 ± 3.76	2.37 ± 0.12

Values are expressed as mean ± SD (*n* = 3). TPC, total phenolic content; TFC, total flavonoid content; HCA, total hydroxycinnamic acid content; TPAC, total proanthocyanidin content; DW, dry weight; GAE, gallic acid equivalents; RE, rutin equivalents; CRE, chlorogenic acid equivalents; EE, epicatechin equivalents.

**Table 5:** Sampling dates, phenological stages, and meteorological conditions recorded during the collection of *R. tomentosum* leaves from October 2023 to October 2024.

Sample Code	Collection Time	Phenological Stage	Average Air Temperature (°C)	Precipitation (mm)	Cloudiness	Relative Humidity
DT1	18 October 2023	autumn vegetative stage	7.8	8.4	69.90%	87.80%
DT2	10 November 2023	autumn vegetative stage	6.5	0	74.40%	84.00%
DT3	5 December 2023	winter dormancy	−2.3	0.2	99.50%	92.80%
DT4	27 December 2023	winter dormancy	2.6	0.6	72.60%	85.80%
DT5	19 January 2024	winter dormancy	−3.7	0	52.80%	87.00%
DT6	8 February 2024	winter dormancy	−1.6	0	88.10%	77.60%
DT7	3 March 2024	winter dormancy	4.7	0	27.50%	68.90%
DT8	18 March 2024	early spring vegetation	1.9	0.1	92.20%	84.80%
DT9	18 March 2024	early spring vegetation	1.9	0.1	92.20%	84.80%
DT10	5 April 2024	early spring vegetation	8.4	5.8	81.70%	85.20%
DT11	18 April 2024	bud formation	6.6	1.2	90.80%	81.10%
DT12	12 May 2024	early flowering	9.5	0	7.90%	54.60%
DT13	23 May 2024	flowering	19.8	0	6.30%	57.00%
DT14	13 June 2024	flowering	19.9	0	47.80%	66.60%
DT15	27 June 2024	fruit development	23.7	0	1.60%	58.80%
DT16	12 July 2024	fruit development	22.4	0.3	53.50%	76.00%
DT17	25 July 2024	fruit development	18.8	21.1	89.70%	87.90%
DT18	20 August 2024	fruit development	20.3	0	41.10%	79.10%
DT19	15 September 2024	autumn vegetative stage	18.1	0	55.60%	84.00%
DT20	10 October 2024	autumn vegetative stage	15.7	0.4	70.30%	86.20%

**Table 6:** Validation parameters of the HPLC–PDA method used for quantification of phenolic compounds in *R. tomentosum* leaf extracts.

Compound	λ (nm)	Linearity Range (µg/mL)	Equation	R^2^	Recovery (%)	LOD (µg/mL)	LOQ (µg/mL)	Precision (% RSD)	
								Intra-Day (*n* = 6)	Inter-Day (*n* = 3)
Neochlorogenic acid	280	1.56–100	y = 42,500x − 15,000	0.999701	97.8	1.36	4.12	0.52	0.89
Chlorogenic acid	280	1.56–100	y = 12,700x − 1330	0.999673	96.9	2.34	7.08	0.61	1.12
Catechin	280	1.56–100	y = 13,000x − 3080	0.999990	98.4	1.00	3.03	0.74	1.05
Fraxetin	360	1.56–100	y = 10,600x − 5180	0.999952	102.3	0.90	2.73	0.68	0.97
Epicatechin	280	1.56–100	y = 12,100x − 3000	0.999972	99.1	0.69	2.08	0.83	1.24
Taxifolin	280	1.56–100	y = 52,700x + 6730	0.999998	101.2	0.11	0.34	0.57	0.94
Procyanidin A1	280	1.56–100	y = 5460x − 3090	0.999698	98.7	2.25	6.82	0.92	1.31
Rutin	360	1.56–100	y = 12,200x − 1860	0.999991	101.4	0.39	1.19	0.71	1.08
Hyperoside	360	1.56–100	y = 18,900x + 644	0.999926	96.6	1.12	3.40	0.64	0.91
Isoquercitrin	360	1.56–100	y = 29,400x + 5180	0.999924	97.9	1.13	3.42	0.88	1.17
Reynoutrin	360	1.56–100	y = 23,800x − 2890	0.999923	98.1	1.14	3.44	0.55	0.86
Guaijaverin	360	1.56–100	y = 22,400x − 3230	0.999994	99.8	0.32	0.96	0.97	1.28
Avicularin	360	1.56–100	y = 11,100x + 2000	0.999927	103.6	1.11	3.36	0.79	1.03
Quercitrin	360	1.56–100	y = 8160x − 2450	0.999958	99.3	0.84	2.54	0.62	0.99
Spiraeoside	360	1.56–100	y = 20,600x − 546	0.999905	97.5	1.26	3.82	0.85	1.14
Quercetin	360	1.56–100	y = 74,800x − 70,300	0.999836	101.7	0.95	2.87	0.46	0.93

## Data Availability

The original contributions presented in this study are included in the article. Further inquiries can be directed to the corresponding author.
